# Pseurotin A Validation as a Metastatic Castration-Resistant Prostate Cancer Recurrence-Suppressing Lead via PCSK9-LDLR Axis Modulation

**DOI:** 10.3390/md21040215

**Published:** 2023-03-28

**Authors:** Khaldoun S. Abdelwahed, Abu Bakar Siddique, Hassan Y. Ebrahim, Mohammed H. Qusa, Ethar A. Mudhish, Ashkan H. Rad, Mourad Zerfaoui, Zakaria Y. Abd Elmageed, Khalid A. El Sayed

**Affiliations:** 1 School of Basic Pharmaceutical and Toxicological Sciences, College of Pharmacy, University of Louisiana at Monroe, Monroe, LA 71201, USA; 2 Department of Surgery, School of Medicine, Tulane University, New Orleans, LA 70112, USA; 3 Department of Biomedical Sciences, Discipline of Pharmacology, Edward Via College of Osteopathic Medicine, University of Louisiana at Monroe, Monroe, LA 71203, USA

**Keywords:** castration-resistant prostate cancer, cholesterol, LDLR, colony formation, fermentation, immunohistoscore, PCSK9, pseurotin A, recurrence, tissue microarray

## Abstract

Metastatic castration-resistant prostate cancer (mCRPC) cells can de novo biosynthesize their own cholesterol and overexpress proprotein convertase subtilisin/kexin type 9 (PCSK9). PCSK9 proved to contribute to mCRPC cell motility since PCSK9 knockdown (KD) in mCRPC CWR-R1ca cells led to notable reductions in cell migration and colony formation. Human tissue microarray results proved a higher immunohistoscore in patients ≥ 65 years old, and PCSK9 proved to be expressed higher at an early Gleason score of ≤7. The fermentation product pseurotin A (PS) suppressed PCSK9 expression, protein–protein interactions with LDLR, and breast and prostate cancer recurrences. PS suppressed migration and colony formation of the CWR-R1ca cells. The progression and metastasis of the CWR-R1ca-Luc cells subcutaneously (sc) xenografted into male nude mice fed a high-fat diet (HFD, 11% fat content) showed nearly 2-fold tumor volume, metastasis, serum cholesterol, low-density lipoprotein cholesterol (LDL-C), prostate-specific antigen (PSA), and PCSK9 levels versus mice fed a regular chow diet. Daily oral PS 10 mg/kg treatments prevented the locoregional and distant tumor recurrence of CWR-R1ca-Luc engrafted into nude mice after primary tumor surgical excision. PS-treated mice showed a significant reduction in serum cholesterol, LDL-C, PCSK9, and PSA levels. These results comprehensively validate PS as an mCRPC recurrence-suppressive lead by modulating the PCSK9-LDLR axis.

## 1. Introduction

Castration-resistant prostate cancer (CRPC) is an aggressive prostate cancer (PC) phenotype with very active progression and a metastatic profile despite androgen deprivation therapy (ADT) and castration levels of testosterone (<0.5 ng/mL) [1]. There are no effective treatment interventions for CRPC. Targeted therapies mostly promote CRPC differentiation to non-treatable neuroendocrine or double-negative phenotypes [2,3]. Relapsed patients show a rise in serum levels of prostate-specific antigen (PSA) and the emergence of locoregional and/or distant tumor recurrences. CRPC was previously named “hormone-refractory prostate cancer” and “androgen-independent prostate cancer” [2,3]. Androgens are essential for the growth and differentiation of prostate cells. Testosterone is the major androgen in the circulation and is bioactivated metabolically to 5α-dihydrotestosterone (DHT) by the stereoselective reduction of its ∆^4,5^ system by 5α-testosterone reductase in prostate tissues. Testosterone and DHT bind to and activate androgen receptors (AR) [4]. ADT loses effectiveness over time. Treated PC patients can still show reliance on androgens for AR activation [5]. CRPC can biosynthesize their needed androgens de novo by utilizing intracellular cholesterol, facilitating their survival, progression and motility [6,7]. The intra-tumoral production of androgens led to the development of androgen pathway inhibitors (APIs), including abiraterone acetate and enzalutamide [8,9]. Enzalutamide lacks the AR agonistic activity at effective doses and does not induce PSA expression or AR nuclear translocation, unlike bicalutamide [10]. It inhibits agonist-induced AR nuclear translocation more effectively than bicalutamide. Abiraterone was originally proposed as a CYP17A1 inhibitor but was shown to be metabolized to a potent AR antagonist, suppressing androgen signaling [11]. While these targeted therapies effectively palliate symptoms and prolong life, metastatic castration-resistant prostate cancer (mCRPC) remains incurable because extended use promotes disease differentiation to non-curable phenotypes. Although the overall incidence of PC has declined, the percentage of patients with metastatic disease at diagnosis has increased over the past decade [12]. Patients with CRPC have a median survival range of 9 to 30 months [5]. When the disease is metastatic (mCRPC), the survival median is shortened to 9 to 13 months. The testosterone level within mCRPC proved to be three-fold higher than the observed level in primary PC [5].

Cholesterol is a steroidal lipid that has physiological functions, including maintenance of membrane structures, signal transduction, and provision of precursors to bile and androgen synthesis [13]. Cholesterol is obtained from two major sources: exogenously from the diet and endogenously via de novo mevalonic acid pathway biosynthesis within cells [13]. Cholesterol hemostasis between its exogenous and endogenous sources is controlled by a complex network of pathways, transporters, and enzymes to maintain the proper intracellular cholesterol level [14]. Tumor cells obtain cholesterol by de novo synthesis using acetyl CoA and/or circulating lipoproteins. Thus, in cancer cells, cholesterol homeostasis enters a state of dysregulation, demonstrated by the accumulation of cholesterol in tumors, which potentially lead to activated tumor cell proliferation. Endogenous cholesterol also provides a precursor for the de novo biosynthesis of androgens, which act as a mitogen and stimulate cell division as in CRPC [15,16].

Low-density lipoprotein (LDL) is one of the prominent lipoproteins, which is mostly produced in the liver and intestine and subsequently released into circulation. LDL interacts with lipoprotein transporters that facilitate the uptake of lipid contents [17]. LDL receptor (LDLR) is one of the major lipoprotein transporters involved in cholesterol influx. LDLR interacts with different donor lipoproteins to uptake cholesterol [18].

Proprotein convertase subtilisin/kexin type 9 (PCSK9) is produced mainly in the liver, brain, and other organs [19]. PCSK9 contributes to hypercholesterolemia since PCSK9 binds to the EGF-A portion of LDLR and enhances its lysosomal degradation [20]. The more PCSK9, the less LDLR, and so higher LDL-C becomes. This is a basic mechanism of PCSK9 in the liver. Meanwhile, in tumors, PCSK9 and LDLR display different roles based on the tumor type. PCSK9 has progressively proven to be a universal target in cancer. PCSK9 deficiency reduces melanoma metastasis to the liver [21]. In lung cancer, PCSK9 was reported as a promising prognostic marker in patients with advanced non-small-cell lung carcinoma [22]. Moreover, PCSK9 inhibition was suggested as a promising way to enhance immune checkpoint therapy for melanoma (B16F10), triple-negative mouse breast cancer (4T1), and colorectal cancer (MC38 and CT26) [23]. Recently, PCSK9 has been reported to promote gastric cancer metastasis [24]. In prostate cancer, PCSK9 was found to be an inducer for PC progression and recurrence [25,26,27,28]. PCSK9 has proven to be a key player in colorectal cancer progression and metastasis by modulating tumor cell EMT and PI3K/AKT signaling, levels of macrophage migratory inhibitory factor, and lactate levels [29].

**Scheme 1:**
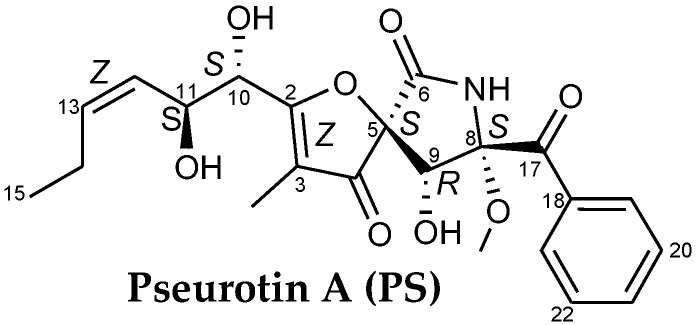
The chemical structure of pseurotin A (PS).

Pseurotin A (PS) (Scheme 1) is a fungal secondary metabolite initially reported from *Pseudeurotium ovalis* strain S2269/F and later identified in several terrestrial *Aspergillus* and *Penicillium* species [20]. PS was among several other secondary metabolites isolated from a cultured *Penicillium* sp. SY2107 isolated from a Mariana Trench sediment specimen collected at −11000 m depth [30]. PS was one of thirty metabolites reported from the fungus *Aspergillus fumigatus* MF071 cultured from the Chinese Bohai Sea sediment [31]. PS was reported as an anti-glioma ingredient of the marine *Bacillus* sp. FS8D [32]. PS was also reported among the bioactive ingredients of the endophytically identified fungal strain *Phoma* sp. NTOU4195 is associated with the marine red alga *Pterocladiella capillacea* [33]. PS was identified among other known metabolites in *Aspergillus niger* species cultures from Brazilian Northeast coast sediments [34]. PS was also reported in several other marine-derived *Aspergillus* species [35]. Chemically, PS has the extremely rare 1-oxa-7-azaspiro-[4,4]non-2-ene-4,6-dione skeleton with 5 chiral centers. PS competitively inhibited fungal chitin synthase, defined as an apomorphine antagonist and food preservative due to its bacteriostatic activity, showed anti-inflammatory activity, inhibited IgE production, inhibited osteoclasts, preventing osteoporosis, and showed anti-seizure activity in animal models [20,35,36]. PS was reported as a novel lead suppressor for the control of breast and prostate cancer progression and recurrence by targeting the PCSK-LDLR axis and regulating tumor cholesterol metabolism [20,36]. PS recently showed a high single-dose safety profile when orally tested in male and female Swiss albino mice at 10, 250, and 500 mg/kg treatment doses and showed an LD_50_ value of >550 mg/kg [37]. The AR in normal prostate epithelial cells act as a tumor suppressor, suppressing these cells’ indefinite proliferation [38]. The AR tumor suppressor function is usually reversed in androgen depletion-independent (ADI) PC. AR is absolutely not expressed in type I ADI PCs represented by PC-3 and DU-145 cells [38]. The reversed AR function, acting as an oncogenic driver, is well presented in the type II ADI mCRPC cells CWR-R1ca [38,39]. The PC-3 and DU145 human prostate cancer cell lines are representative of the earlier type I ADI prostate cancers. An earlier study documented the PCSK9-LDLR axis-driven suppressive activity of PS against the type I ADI PC cell line PC-3 [36]. The current study provides comprehensive evidence for the role of PCSK9 in PC pathogenesis as well as the lead validation of PS as a potent recurrence-suppressing entity for the more aggressive and metastatic type II ADI PC represented by CWR-R1ca [39] by targeting the PCSK9-LDLR axis.

## 2. Results

### 2.1. CWR-R1ca Cells Demonstrated Significant PCSK9 Axis Dysregulation

**Figure 1:**
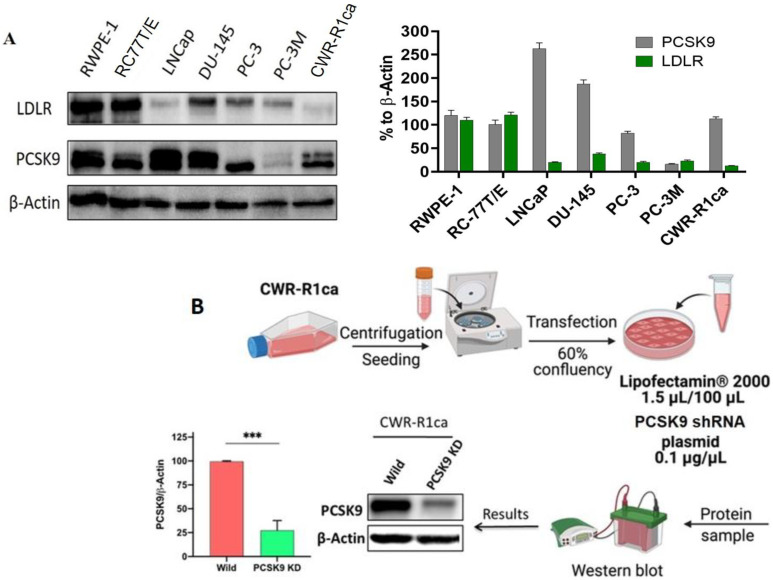
Western blot analysis of PCSK9 and LDLR expression in PC cell lines and overview and validation of PCSK9 knockdown in CWR-R1ca cells. (**A**) PCSK9 and LDLR expression levels in diverse PC cell lines (left panel). Densitometric analysis of PCSK9 and LDLR expression levels (right panel). (**B**) Overview of methodology and validation of the knockdown efficacy of PCSK9 in CWR-R1ca cell transfection using shRNA-plasmid specific to PCSK9. Western blotting showed a 75% PCSK9 reduction in transfected CWR-R1ca-KD cells compared to the wild-type cells. *** *p* < 0.01 for statistical significance. Scanning densitometry was obtained for all blots, carried out in duplicate, and the integrated optical density of each band was normalized with the corresponding density found for β-actin in the same blot. Results are shown in the bar graphs to their respective Western blot images. Vertical bars in the graph indicate the normalized integrated optical density of bands visualized in each lane.

To investigate the relevance of PCSK9 in PC, we evaluated PCSK9 and LDLR expression levels by Western blot in diverse stages of a PC cell panel (Figure 1). This included LNCaP (androgen-dependent), the type I ADI DU-145, PC-3 and PC-3M PC cell lines, which lack AR expression and CWR-R1ca cells (castration-resistant, type-II ADI, which overexpress both wild and splice variant 7 AR) [39]. The results showed diverse ratios of PCSK9-LDLR expression among tested PC cells (Figure 1A). The mCRPC CWR-R1ca cell line was chosen for this study since it represents one of the most highly aggressive and metastatic type II ADI PC phenotypes and has a highly dysregulated PCSK9-LDLR ratio (Figure 1A).

### 2.2. PCSK9 Knockdown in CWR-R1ca Cells

To study the role of PCSK9 in CRPC, we knocked down the PCSK9 gene in CWR-R1ca cells using PCSK9 shRNA-lipofectamine (Figure 1B). Western blotting of PCSK9 level was used as an indicator of KD efficiency. CWR-R1ca PCSK9-KD cells showed ~75% reduction in PCSK9 expression compared to the wild-type cells (Figure 1B).

### 2.3. PCSK9 Knockdown Did Not Notably Affect CWR-R1ca Cells’ Proliferation

**Figure 2:**
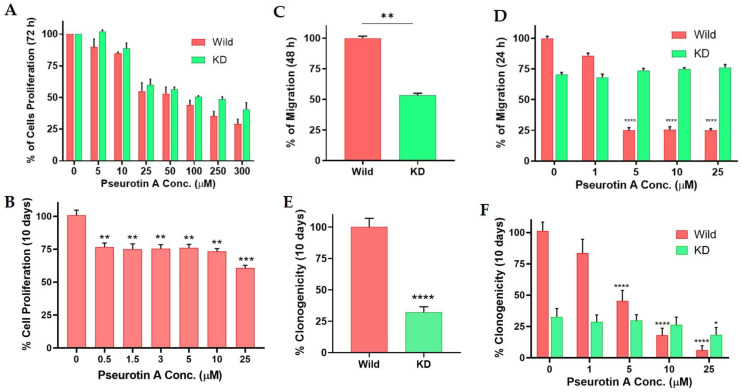
Effects of PS and PCSK9 knockdown on the proliferation, migration, and colony formation of CWR-R1ca cells. (**A**) Comparison of different PS treatments on the proliferation of wild and PCSK9-KD CWR-R1ca cells over 72 h culture period. (**B**) Long-term (10-day) anti-proliferative effects of PS on wild-type CWR-R1ca cells. Vertical bars indicate mean cell count ±SEM (*n* = 6) in MTT/proliferation assay for each treatment group over the same culture period. (**C**) Comparison of the migratory ability of wild and PCSK9-KD CWR-R1ca cells in wound-healing scratch assay. (**D**) Comparison of the antimigratory effect of PS treatments against the wild-type versus PCSK9-KD CWR-R1ca cells over 24 h. (**E**) Comparison of the wild-type versus PCSK9-KD CWR-R1ca cells’ colony formation ability. (**F**) Comparison of PS treatments’ anti-clonogenicity activity in wild-type versus PCSK9-KD CWR-R1ca cells over 12 days. Vertical bars indicate the percentage of cell migration and colony formation of wild-type and CWR-R1ca-KD cells at the experiment end calculated relative to the vehicle-treated controls (*n* = 3). * *p* < 0.05, ** *p* < 0.01, *** *p* < 0.001, and **** *p* < 0.0001 for statistical significance compared to VCs.

We investigated the effect of PCSK9 KD on the CWR-R1ca cells’ viability using an MTT assay. Results showed that PCSK9 KD has no statistical proliferation suppression over 72 h in comparison to wild-type CWR-R1ca cells (Figure 2A). Different PS treatments showed no statistical concentration-dependent proliferation inhibition over 72 h in both wild and PCSK9-KD CWR-R1ca cells (Figure 2A). PS showed no cytotoxic activity against CWR-R1ca cells in a long-term cell viability study, suggesting that PS acts as a cytostatic agent rather than as a cytotoxic agent [40]. CWR-R1ca cell viability monitoring over 10 days showed a consistent number of viable cells at different PS treatment doses (Figure 2B) [40].

### 2.4. PCSK9 Knockdown Suppressed the CWR-R1ca Cells’ Migration Activity

Cell migration is among the early steps in the metastatic tumor cascade. We investigated the role of PCSK9 KD on CWR-R1ca cells’ migration using the wound-healing assay. Results showed that PCSK9 KD notably suppressed the migration ability of CWR-R1ca cells. CWR-R1ca-KD cells lost ~46.7% of their migratory ability in comparison to the wild-type cells (Figure 2C). This finding indicated that PCSK9 could be critical for the migration of CWR-R1ca cells. Different PS treatment doses (1–25 µM) were used to assess their anti-migratory efficacy against the wild-type CWR-R1ca and CWR-R1ca-KD cells over 24 h after cells were scratched. Results showed the PS anti-migratory activity against wild CWR-R1ca cells, with an IC_50_ of 2.5 µM (Figure 2D). PS treatments had no effect on the migration of the CWR-R1ca-KD cells (Figure 2D). These findings demonstrated that PS dose-dependently and selectively suppressed the wild-type CWR-R1ca cells’ migratory activity, unlike its effects against the CWR-R1ca-KD cells, suggesting that targeting PCSK9 in CRPC may notably diminish its migratory ability.

### 2.5. PS Suppressed CWR-R1ca Cells’ Clonogenicity

A colony formation assay was performed to study the contribution of PCSK9 on the clonogenicity of the CRPC CWR-R1ca cells and to assess the anti-clonogenicity activity of PS against wild-type CWR-R1ca and CWR-R1ca-KD cells. The results showed that PCSK9 KD significantly reduced the clonogenic survival of CWR-R1ca cells by ~67.4% (Figure 2E). Furthermore, PS was more effective against the colonization of the wild-type CWR-R1ca cells compared to CWR-R1ca-KD cells. The IC_50_ of PS anti-clonogenicity activity was 4.3 µM against wild-type CWR-R1ca cells, while it was higher than 25 µM against CWR-R1ca-KD cells (Figure 2F). A 5 µM, PS allowed only 45.3% clonogenicity in wild-type CWR-R1ca cells, while 25 µM PS treatment allowed 56.3% clonogenicity in CWR-R1ca-KD cells, implying the significant contribution of PCSK9 to the anti-clonogenic effect of PS. The clonogenicity response of PS against CWR-R1ca-KD cells may be attributed to the remaining ~25% of PCSK9-containing cells since the PCSK9 knockdown achieved ~75% success in CWR-R1ca-KD cells.

### 2.6. TMA Immunohistochemistry

**Figure 3:**
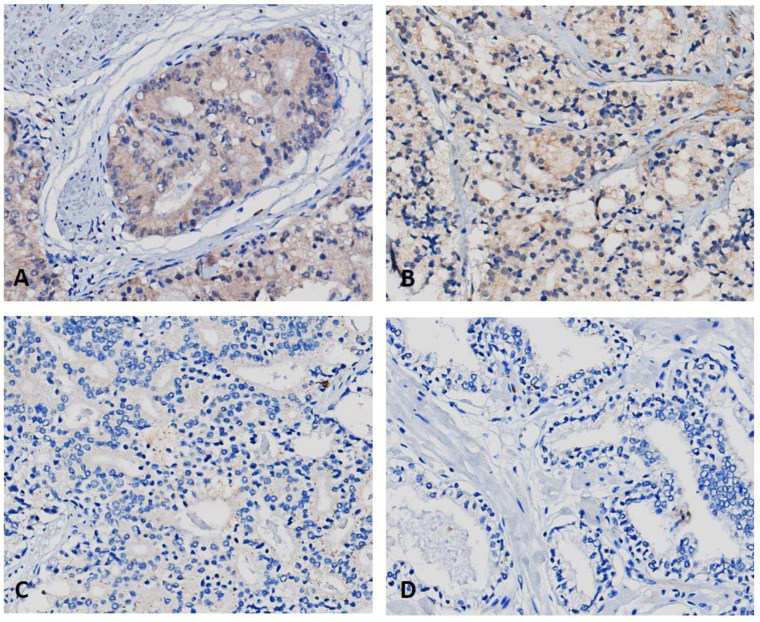
Expression of PCSK9 in PC tissue cores. PCSK9 expression was evaluated by immunohistochemistry in tissue microarray slide comprising 64 cases/192 PC tissue cores and 6 normal prostate cases/18 tissue cores. The protein expression was evaluated in tissues collected from PC patients at Gleason score 5 (**A**), GS = 7 (**B**), and GS = 8 (**C**) in addition to normal prostate tissues (**D**). Magnification is 400×.

**Figure 4:**
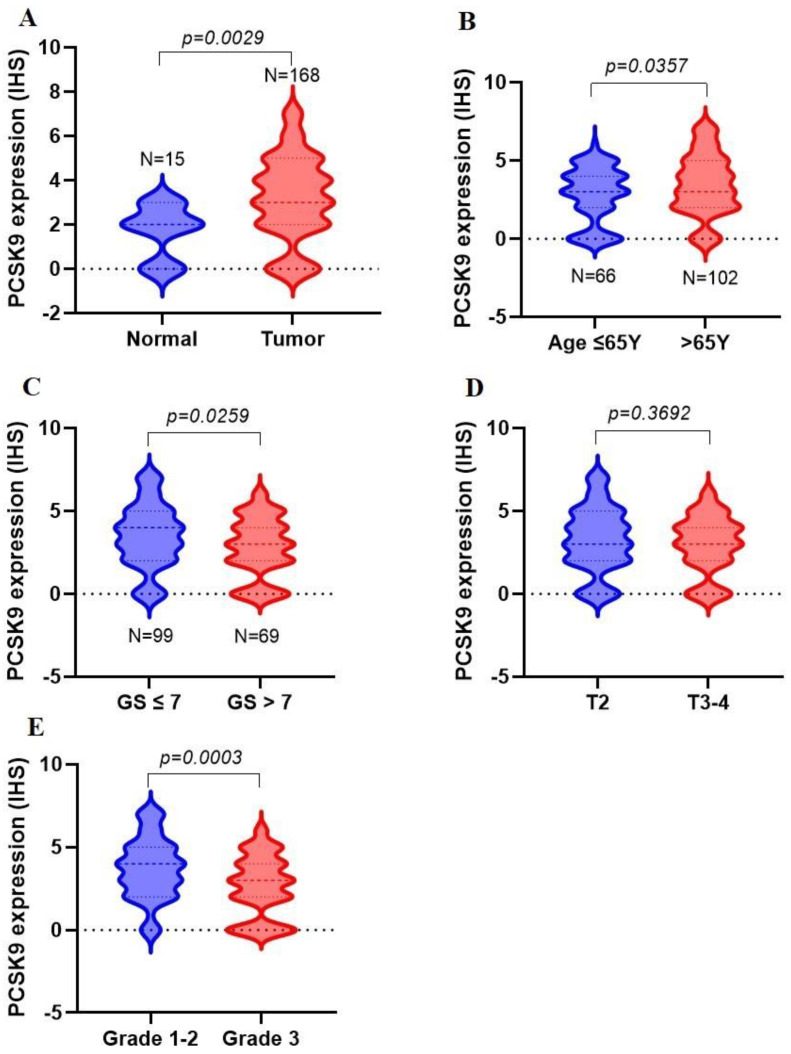
PCSK9 expression in PC tissues expressed as immunohistochemical score (IHS). PCSK9 expression in PC tissue cores was considered regarding normal tissue cores (**A**), age at diagnosis (**B**), Gleason score (GS) (**C**), pathological stages (T2–4) (**D**) and tumor grades (1–3) (**E**). Unpaired *t*-tests were performed, and significance of the data was calculated at *p* < 0.05.

Before staining the TMA tissue slide, we used three PC tissue slides for antibody optimization; two of them were stained with low and high concentrations of the antibody, and the third one was incubated with the secondary antibody only as a negative control. The results showed a cytoplasmic signal at a dilution of 1:500 and with no signal in the negative control slide. The available clinical information of PC patients and non-PC individuals is provided in Table S1 (Supplementary Material). Immunohistochemical staining of PCSK9 showed a higher protein expression in the cytoplasm of PC tissues (*p* = 0.0029) compared to the normal tissues (Figure 3). A higher immunohistoscore (IHS) was observed for PCSK9 expression in patients aged 65 years old or older (*p* = 0.0357) versus younger patients. When the Gleason score (GS) was adjusted, PCSK9 expression was higher in patients with an early GS of ≤7 (*p* = 0.0259), and the signal was slightly decreased at a higher GS (Figure 3A–C). There was no significant difference in the protein expression in tissues collected from patients at low or high pathological stages (*p* = 0.3692). However, tumor grades 1–2 showed an increase in PCSK9 signal (*p* = 0.0003) compared to tumor grade 3 (Figure 4).

### 2.7. High Fat Diet-Induced CWR-R1ca CRPC Progression and Metastasis in a Nude Mouse Xenograft Model

**Figure 5:**
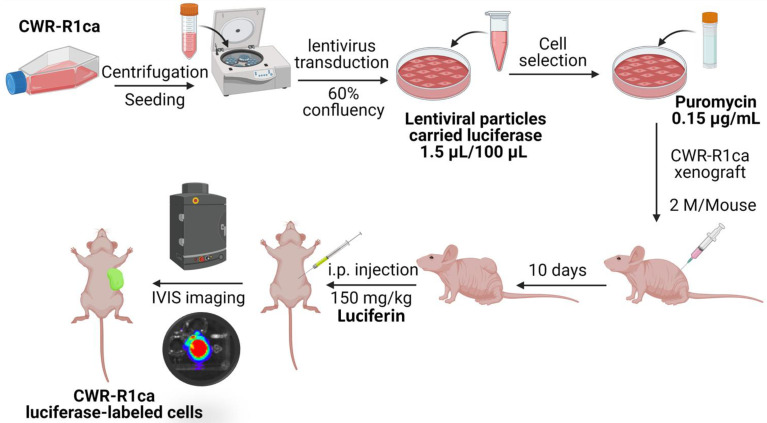
CWR-R1ca cells transduction using lentivirus-carried luciferase particles.

**Figure 6:**
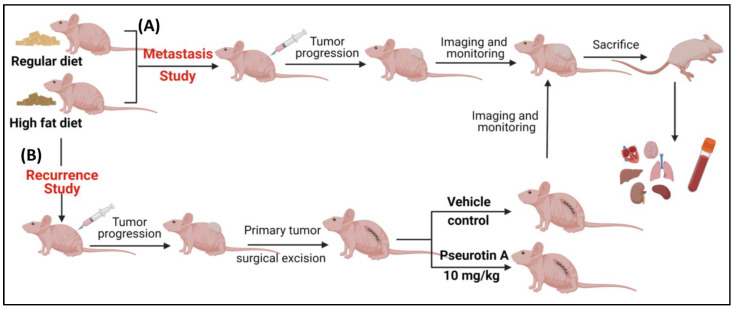
Overview of animal studies design using CWR-R1ca-Luc cells. (**A**) Comparison of the impact of HFD versus regular diet on the progression and metastasis of CWR-R1ca-Luc cells in a xenograft nude mouse model. (**B**) Recurrence of suppressive effects of PS against the CWR-R1ca-Luc cells.

**Figure 7:**
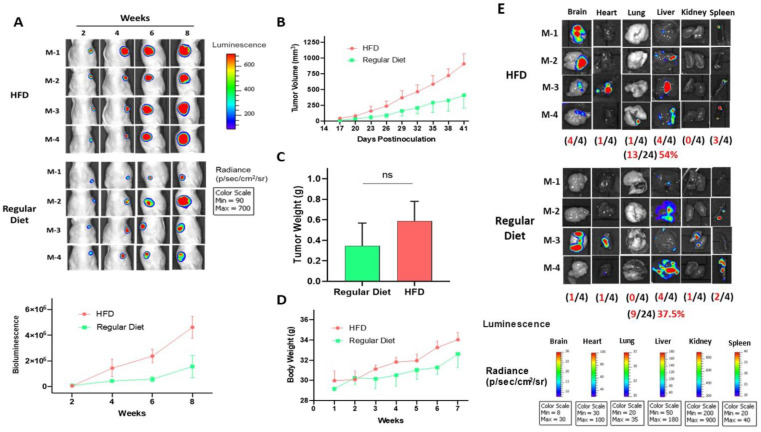
Effect of HFD on the progression and metastasis of the mCRPC CWR-R1ca-Luc cells xenografted into nude mice. (**A**) Biweekly live animal monitoring of CWR-R1ca-Luc cell bioluminescence. The upper panel shows the comparison of CWR-R1ca-Luc cells’ bioluminescence progression of individual mice in each of HFD- and regular diet-fed groups. The lower panel represents bioluminescence intensity monitoring in intact animals over the experiment course. (**B**) Comparison of the mean tumor volumes of HFD versus regular diet over the experiment course. Bars represent the mean tumor volume; error bars represent the SEM for each experimental group. (**C**) Comparison of the mean tumor weights for HFD-fed versus regular diet-fed mice at the experiment end. Error bars indicate SEM. (**D**) Mice body weight-monitoring data over the experiment course. Points represent the mean body weight of animals in each group. Error bars indicate SEM. (**E**) Bioluminescence and morphological comparison of collected organs from the HFD-fed (upper panel) versus regular diet-fed mice group (lower panel) at the end of the study. “ns” consider statistically not significant *p* > 0.5.

The luciferase-tagged CRPC CWR-R1ca-Luc cells were used to generate a bioluminescent xenograft model (Figure 5). This enabled the live animal monitoring of tumor metastasis to bone, kidney, liver, lung and brain. This model was used to study the in vivo effect of HFD on the CRPC progression and metastasis to distant organs (Figure 6). Male athymic nude mice were fed an HFD or regular diet for 10 days before tumor cell engrafting. CWR-R1ca-Luc cells (2 × 10^6^ cells per mouse) were inoculated into the suprascapular region. Upon tumor formation, mice bioluminescence was imaged weekly over the experimental period to monitor the tumor progression and metastasis (Figure 6). Bioluminescence imaging showed the enhanced HFD induction ability for tumor progression, evidenced by the increase in the tumor volume intensity in comparison to a regular diet over the study course (Figure 7A). Physically, tumor volumes were measured using a digital caliber over the study course. HFD-fed mice showed significant tumor growth, with a mean tumor volume of 906.4 ± 139.8 mm^3^ in comparison to regular-diet-fed mice, with a mean tumor volume of 401.8 ± 177.8 mm^3^, representing a 2.3-fold induction of tumor growth (Figure 7B). The mean tumor weight was 0.59 ± 0.16 g and 0.35 ± 0.19 g for mice fed an HFD and regular diet, respectively, representing a 1.7-fold increase in tumor weight (Figure 7C). Mice body weight was monitored over the study course and showed that an HFD increases mice body weight in comparison to a regular diet (Figure 7D). Bioluminescence imaging of collected mice organs at the study end proved that an HFD significantly induced tumor metastasis to organs in comparison to the regular diet group. The HFD-fed group showed metastasis in 54% of the collected animal organs in comparison to 37.5% in the regular diet-fed group (Figure 7E). The brain and liver were the most targeted organs by tumor cell metastasis.

CWR-R1ca-Luc cell transduction efficiency was tested in a xenografted mouse model using a PerkinElmer (Waltham, MA, USA) IVIS imaging system.

### 2.8. Effect of HFD on the Total Serum Cholesterol and LDL-C Levels

**Figure 8:**
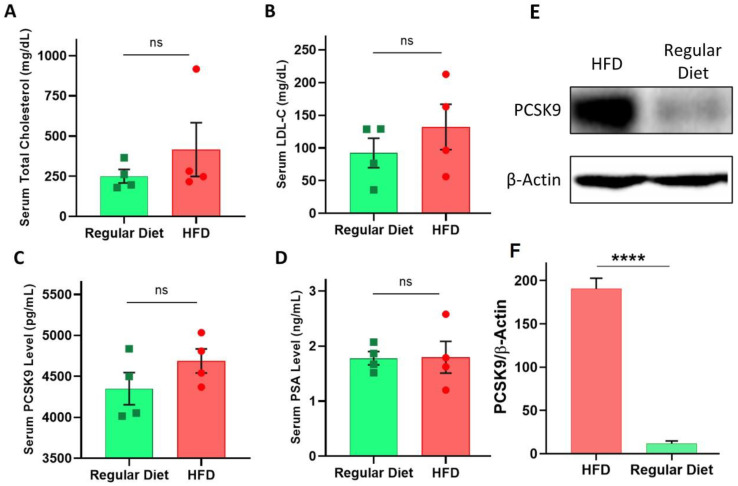
Systemic effects of HFD versus regular diet on mice serum cholesterol, LDL-C, PCSK9, and PSA levels. (**A**) Comparison of the HFD’s effect versus regular diet on mice mean serum total cholesterol levels. (**B**) Comparison of the HFD’s effect versus regular diet on mice serum LDL-C levels. (**C**) Comparison of the HFD’s effect versus regular diet on mice serum PCSK9 levels. (**D**) Comparison of the HFD’s effect versus regular diet on mice serum PSA levels. (**E**) Comparative of Western blotting analysis of HFD’s effect versus regular diet on the PCSK9 expression levels in CWR-R1ca cell tumors. (**F**) Western blot densitometric analysis of PCSK9 expression levels. Error bars represent the SEM for each experimental group. Each point is the mean of triplicate. **** *p <* 0.0001 for statistical significance compared to regular diet group. “ns” consider statistically not significant *p* > 0.5.

Mice fed an HFD over the experiment duration displayed an increase in their cholesterol profile with an average of 416.12 ± 155.9 mg/dL and 132.08 ± 30.1 mg/dL for total cholesterol and LDL-C, respectively, in comparison to mice fed a regular diet, which had an average of 250.87 ± 22.9 mg/dL and 92.42 ± 19.5 mg/dL for total cholesterol and LDL-C levels, respectively (Figure 8A,B).

### 2.9. Effect of HFD on Serum PCSK9 Levels

The HFD-fed group of mice showed increased serum PCSK9 levels in comparison with mice fed a regular diet. The mean serum PCSK9 levels were 4688.6 ± 126.9 pg/mL and 4350.9 ± 169.4 pg/mL for the HFD- and regular diet-fed mice, respectively (Figure 8C).

### 2.10. Effect of HFD on Serum PSA Levels

HFD supplementation did not affect PSA levels significantly in comparison to mice fed a regular diet; the mean serum PSA levels were 1.80 ± 0.24 ng/mL and 1.78 ± 0.10 ng/mL for the HFD and regular diet, respectively (Figure 8D). Thus, the elevated fat diet did not confer changes in serum PSA levels. Subsequent in vivo studies should be designed to observe tumor recurrence in mice fed an HFD.

### 2.11. Effect of HFD on PCSK9 Expression Levels in Nude Mice CWR-R1ca-Luc Tumors

Although the HFD-fed mice did not show a significant increase in serum PCSK9 levels in comparison with mice fed a regular diet, Western blot analysis of collected CWR-R1ca-Luc tumors showed a significant increase in PCSK9 expression levels in tumors collected from HFD-fed mice, unlike the tumors collected from mice fed the regular diet. The HFD showed 75% higher PCSK9 expression levels compared to the tumors from the group of mice fed a regular diet (Figure 8E,F).

### 2.12. Pseurotin A Daily Oral 10 mg/kg Significantly Suppressed CWR-R1ca-Luc Tumor Locoregional and Distant Recurrences

**Figure 9:**
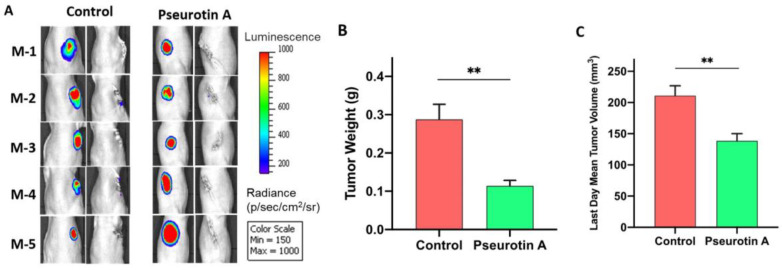
Surgical excision of CWR-R1ca-Luc mCRPC primary tumor. (**A**) Bioluminescence imaging of individual intact mice validated the efficacy of CWR-R1ca-Luc primary tumor surgical excision. (**B**) Comparison of the mean tumor weight of PS-treated mice (10 mg/kg for one week before surgery) versus vehicle-treated control mice. (**C**) Comparison of the last day mean tumor volume of PS-treated mice versus vehicle-treated control. Error bars represent the SEM for each experimental group. ** *p* < 0.01, for statistical significance.

Since PS showed potent in vitro motility-suppressive activities and lacked anti-proliferative activity, the in vivo recurrence suppressive effects of PS were examined in the most aggressive mCRPC CWR-R1ca cells in a nude xenograft model. HFD-fed mice were injected with CWR-R1ca-Luc cells 2 × 10^6^ into their suprascapular region. Daily oral PS 10 mg/kg gavage dosing started one week before the primary tumor resection surgery and continued for an additional eight weeks. Mouse tumors were surgically excised when each tumor volume reached 200 mm^3^ in the VC group. Bioluminescence imaging immediately after primary tumor excision surgery was used to confirm efficient tumor surgical excision (Figure 9A) [41,42]. The mean primary tumor weight was 0.11 ± 0.01 g for the PS and 0.28 ± 0.03 g and VC-treated groups, representing a 60.7% tumor weight reduction (Figure 9B). The PS-fed mice showed a 34.3% reduction in the mean primary tumor volume, 138.7 ± 10.3 mm^3^, as compared to the VC-treated mice tumor volume of 211 ± 14.2 mm^3^ (Figure 9C).

**Figure 10:**
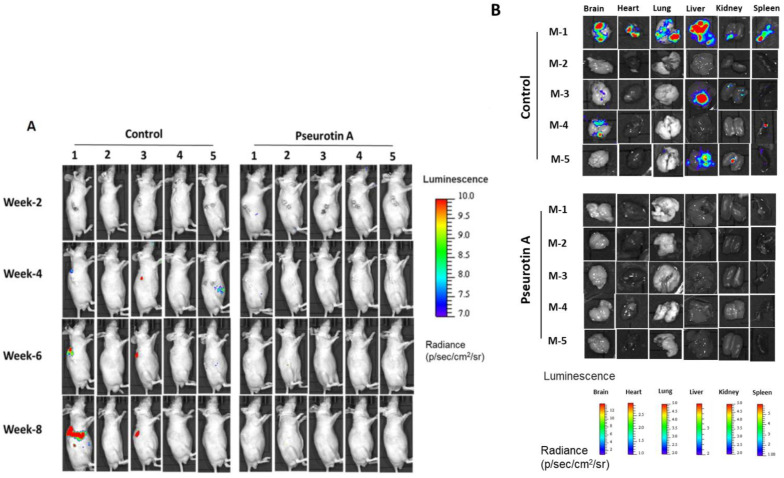
Effect of PS daily oral 10 mg/kg treatments on locoregional and distant recurrences of CWR-R1ca-Luc mCRPC cells after primary tumor surgical excision. (**A**) Biweekly CWR-R1ca-Luc cell bioluminescence monitoring in live mice over the eight-week recurrence study. (**B**) Bioluminescence and morphological comparison of animal organs for PS-treated mice versus VC-treated animals collected at the study end. PS-treated mice showed neither locoregional nor organ-distant recurrences in comparison to VC group.

The PS effects on locoregional and distant tumor recurrences study was conducted by continued dosing of mice subjected to primary tumor surgical excision for an additional eight weeks after surgery. Weekly bioluminescence imaging was used to monitor tumor recurrence [41,42]. Live animal bioluminescence imaging showed the persistent ability of PS treatments to effectively inhibit mCRPC locoregional and distant recurrences over the study course (Figure 10A). When the study was terminated, the bioluminescence imaging of collected mouse organs proved that PS treatments significantly prevented distant tumor recurrence to the brain, lung, heart, liver, kidney, and spleen in comparison with the VC-treated animals (Figure 10B). None of the PS-fed mice had any organ tumor distant recurrences, unlike the vehicle-treated mice, which showed four out of five mice with distant tumor recurrence in different organs (Figure 10B).

### 2.13. Pseurotin A Reduced Nude Mice Serum Total Cholesterol and LDL-C Levels

**Figure 11:**
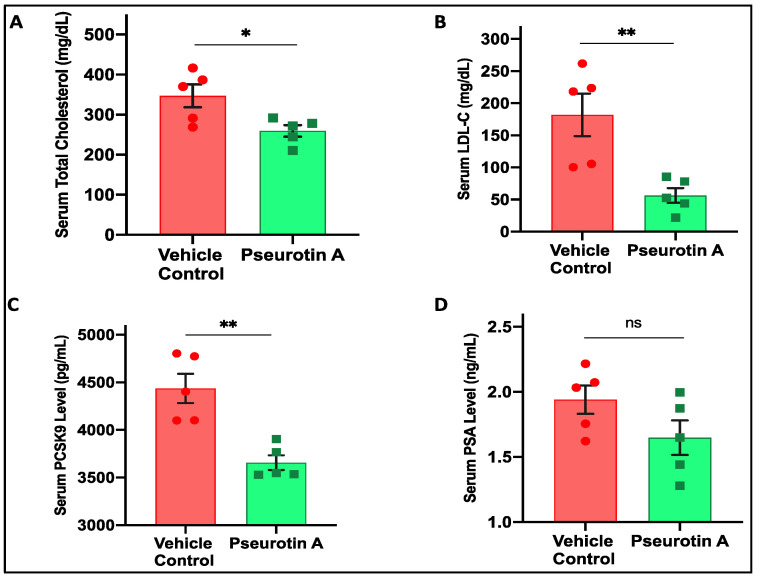
Systemic effects of PS treatments on mice serum cholesterol, LDL-C, PCSK9, and PSA levels. (**A**) Comparison of PS treatment effects on total serum cholesterol levels versus VC group. (**B**) PS versus VC treatment effects on mice mean serum LDL-C levels. (**C**) PS versus VC treatment effects on mice mean serum PCSK9 levels. (**D**) Treatment effects of PS versus VC on mice mean serum PSA levels. Error bars represent the SEM for each experimental group. Each point is the mean of triplicate. * *p* < 0.05, ** *p* < 0.01, for statistical significance. “ns” consider statistically not significant *p* > 0.5.

PS treatments significantly decreased the nude mice’s total serum cholesterol levels, with an average cholesterol level of 259.5 ± 12.8 mg/dL in PS-treated mice compared to 346.6 ± 25.4 mg/dL in VC-treated mice, representing a 25.1% reduction in cholesterol level (Figure 11A). Similarly, PS treatments also significantly decreased the serum LDL-C levels, with an average of 56.4 ± 10.3 mg/dL versus 105.4 ± 29.6 mg/dL for VC-treated mice, representing a 46.4% reduction in LDL-C level (Figure 11B).

### 2.14. Pseurotin A Reduced Nude Mice Serum PCSK9 Levels

Consequent to the reduction of total cholesterol and LDL-C serum levels, PS treatments significantly decreased serum PCSK9 levels in nude mice, with an average of 3657.0 ± 96.4 pg/mL, compared to VC-treated mice with an average of 4436.3 ± 211.7 pg/mL, representing a 17.6% reduction in PCSK9 levels (Figure 11C).

### 2.15. Pseurotin A Reduced Nude Mice Serum PSA Levels

PS treatments decreased the serum PSA level, with an average of 1.64 ± 0.17 ng/mL in PS-treated mice compared to 1.95 ± 0.13 ng/mL in VC-treated mice, which represented a 15.9% reduction in PSA level (Figure 11D).

## 3. Discussion

Although several cytotoxic and targeted therapeutic options are available for CRPC, effective recurrence inhibitors are lacking. The extended use of androgen pathway inhibitors such as enzalutamide lack curative efficacy and can actually promote the differentiation of CRPC to incurable phenotypes [2,3,9,10,11,12]. Current therapies have limited disease recurrence preventive potency, and most therapies fail to eradicate residual quiescent tumor cells [43]. Treatment delays in PC can also enhance the recurrence risk of PC [44]. Epidemiological studies shed light on the role of cholesterol in PC incidence and progression [45]. Several reports suggested that hypercholesterolemia is associated with an increased risk of aggressive metastatic PC [46,47]. The mechanistic and translational understanding of the potential roles of cholesterol and the PCSK9 axis in mCRPC is poorly understood [36,45,46,47,48]. The discovery of de novo androgen biosynthesis in advanced metastatic PC proved a potential mechanism in which prostate tumors escape the dependence on circulating androgens [6,48,49]. Thus, ADT palliative efficacy is lacking. Recurrent PC tissues express high levels of AR and AR-regulated genes, which suggests their relevant role in PC recurrence [49]. Novel therapies are direly needed to modulate AR directly or its endogenous androgen ligand’s biosynthesis, preventing its activation within PC cells. This is specifically important in type II ADI CRPC, which usually expresses high level of AR [2,3,4,5,38,39]. PCSK9 is a serine protease protein expressed mainly in the liver, brain, intestine, and other organs [20,21,22,23,24,29,36]. It acts as a modulator of intracellular and plasma cholesterol homeostasis [50,51,52]. PCSK9-LDLR axis is responsive to variations in cellular cholesterol levels through sterol regulatory element-binding protein 2 [53,54] and hepatocyte nuclear factor 1 [54]. Targeting intracellular cholesterol, which is the biosynthetic precursor for androgens, could be achieved by targeting the PCSK9-LDLR axis with small molecules. No previous attempts explored the role of the PCSK9-LDLR axis in CRPC. A recent drug target Mendelian randomization study evaluated the correlation between genetically proxied inhibition of LDL-C lowering drug targets on PC risk [55]. The genetically proxied inhibition of PCSK9 proved to be associated with PC and reduced total and early-onset risks [55]. Unlike PCSK9, there was no association between the genetically proxied HMG-CoA reductase nor the cholesterol transporter NPC1L1 with reduced PC risk, suggesting the important oncogenic role of PCSK9 in PC [55]. The PCSK9 mRNA and protein levels recently proved elevated in gastric tumor patients’ tissues and sera versus healthy subjects [24]. PCSK9 promoted gastric cancer invasion and migration and suppressed apoptosis but did not affect tumor proliferation. PCSK9 silencing in gastric cancer reversed these effects and suppressed metastasis. PCSK9 mediated its gastric cancer-promoting effects by upregulating HSP70 and MAPK signaling [24]. The role of PCSK9 in enhancing hepatocellular tumors and melanoma metastasis has been validated [21,56]. PCSK9 genetic deletion or using PCSK9 antibodies enhanced the major histocompatibility protein class I expression on the tumor cell surface, enabling CD8 T cells to identify cancer cells and improving the cytotoxic T cells’ intratumoral infiltration [23]. PCSK9-LDLR axis dysregulation is a new key player mitogen in multiple malignancies.

Pseurotin A (PS) is a terrestrial and marine fungal secondary metabolite reported to target hypercholesterolemia and hypercholesterolemia-induced breast and prostate cancer recurrence through the suppression of PCSK9 secretion and PCSK9-LDLR PPI [20,36]. Since mCRPC relies on de novo synthesis of cholesterol [48], PS showed promise to reduce this PC aggressive phenotype recurrences as a small molecule that can act intracellularly on the PCSK9-LDLR axis and extracellularly, unlike the FDA-approved mAbs, which act only at the extracellular level because they lack the ability to enter PC cells due to their large molecular size [20,23,36]. PS is expected to control the PC intracellular de novo cholesterol level by modulating the PCSK9-LDLR axis.

Screening of diverse PC cell lines panel revealed the highest PCSK9, and very low LDLR levels were observed in the AR-rich LNCaP PC cells (Figure 1). These cells are highly hormone-dependent, representing early-stage adenocarcinoma, which can be controlled with ADT. Extended exposure to APIs such as enzalutamide promotes the differentiation of the primary prostate adenocarcinoma to the more resistant phenotype CRPC, which has limited therapeutic interventions [3,11]. The PCSK9-LDLR axis was also dysregulated in the type II ADI mCRPC cells, CWR-R1ca cells (Figure 1) [39]. PCSK9-LDLR levels were lowest in the type-I ADI PC-3M cells. PCSK9 and LDLR expression levels in the non-tumorigenic prostate epithelial cells RWPE-1 and the premalignant prostate cells RC77T/E were nearly equal (Figure 1). This was consistent with TMA results in which PCSK9 proved higher in the lower grades and GS in human PC patients (Figure 3 and Figure 4). Early adenocarcinoma may have more need for PCSK9 and cholesterol compared to advanced pathological stages.

The fibroblast-free type-II ADI mCRPC cells CWR-R1ca were selected for this study due to their dysregulated PCSK9 (high) versus low LDLR level and very high recurrence profile [39,43]. To investigate the role of PCSK9 in mCRPC, we knocked down PCSK9 in CWR-R1ca cells (CWR-R1ca-KD). CWR-R1ca-KD cells showed significantly reduced migratory and colony formation patterns compared to the wild-type CWR-R1ca cells (Figure 2). Meanwhile, CWR-R1ca-KD cell proliferation was not statistically different from the wild-type cells, suggesting a clear notable PCSK9 contribution to the motility but not to the tumor progression and survival (Figure 2). Results were consistent with the literature, indicating that PCSK9 gene knockout did not affect tumor growth in NCG mice [23]. In vitro, PS treatments showed dose-dependent migratory and colony formation inhibition activities against wild-type CWR-R1ca cells, unlike its weaker effects against CWR-R1ca-KD cells. PS proliferation inhibitory activity was not statistically significant between both cells. Long-term (10-day) proliferation assays indicated the cytostatic rather than cytotoxic PS effects on wild-type CWR-R1ca cells [40]. The results clearly validated PS as a selective modulator for PCSK9 in wild-type CWR-R1ca cells, and its activity was nearly lost when PCSK9 was knocked down in CWR-R1ca-KD cells.

It is well-established that high-fat-containing diets promote PC progression [45,46,47,48]. In this study, we investigated the role of HFD on the progression and metastasis of bioluminescent-tagged mCRPC CWR-R1ca cells engrafted in nude mice. Mice fed an HFD (11% fats) had nearly double the tumor progression and metastasis to distant organs as compared to mice fed the regular diet (5% fats). The HFD-fed group showed notable induction of serum cholesterol, LDL-C, PCSK9, and PSA versus the regular chow diet group mice (Figure 7). Although the systemic effect of HFD on PCSK9 was not significant, Western blot analysis of collected tumors showed significant induction of intracellular PCSK9 expression in the HFD group mouse tumors compared to the regular diet group. These results demonstrated the mitogenic role of the HFD in mCRPC, which is associated with promoting intracellular PCSK9 expression. Microenvironment interfacing tumor cells and specific organs play a vital role in organ-specific metastasis [23,57,58]. Circulating tumor cell(s) travel to distant organs, where they initiate new tumors when the microenvironment is favorable for invasion and subsequent colonization [57,58]. CWR-R1ca cells showed organ-specific metastasis patterns. Collected organs at the end of the study showed CWR-R1ca tumor cells metastasized and localized mostly in the liver, brain, and spleen (Figure 10).

The type II ADI CWR-R1ca cells provide a good in vivo model to study tumor metastasis since cells of this line express AR, AR-splice variant-7 and PSA [39]. The CWR-R1ca cells have high tumorigenic and recurrent activity. Daily oral PS 10 mg/kg for 7 days notably suppressed the primary tumor progression. Continued PS treatments for eight weeks effectively prevented locoregional and distant CWR-R1ca tumor recurrence after primary tumor surgical excision. The PS recurrence preventive potency was associated with a significant reduction in systemic mice cholesterol and LDL-C levels, suggesting effective pharmacodynamics (Figure 10 and Figure 11). PS treatments also reduced mice serum levels of PCSK9 and the CRPC biochemical recurrence marker PSA. The recently reported high safety profile in Swiss albino mice [31] further validated the lead candidacy of PS as a prospective novel CRPC recurrence suppressive entity. Together, the PCSK9-LDLR axis is a novel molecular target in PC pathogenesis. The novel marine and terrestrial fungal fermentation product PS is a valid orally active dual PCSK9 expression and PPI inhibitor appropriate for application to control PC recurrence.

## 4. Materials and Methods

### 4.1. Chemicals, Reagents and Antibodies

Pseurotin A (PS) was isolated by fermentation of the fungus *Aspergillus fumigatus* [20]. The fermentation, extraction, purification and spectroscopic identity were described previously [20]. A PS purity of >98% was established based on HPLC and q^1^H NMR spectral analysis. All chemicals purchased from Sigma-Aldrich (St. Louis, MO, USA) unless otherwise stated. Cell culture reagents were obtained from Life Sciences (Carlsbad, CA, USA). All antibodies were purchased from Proteintech Group (Rosemont, IL, USA) and used at a dilution of 1:1000 unless otherwise stated.

### 4.2. Cell Lines and Culture Conditions

The human PC cell lines LNCaP (androgen-dependent), the type-I ADI DU-145, PC-3 and PC-3M, along with the non-tumorigenic cell line RWPE-1 were purchased from the American Type Culture Collection (ATCC, Rockville, MD, USA). The premalignant epithelial cells RC77T/E were kindly provided by Dr. J. Rhim, Uniformed University. The type II ADI CWR-R1ca (castration-resistant) cell line was purchased from Millipore-Sigma (Burlington, MA, USA). Cells were cultured in Roswell Park Memorial Institute (RPMI-1640). Cells were supplemented with 10% fetal bovine serum (FBS), penicillin G (100 U/mL), and streptomycin (100 ng/mL) and were maintained in a humidified incubator at 37 °C with 5% CO_2_. For sub-culturing, cells were washed with Ca^2+^- and Mg^2+^-free phosphate-buffered saline (PBS) and incubated in 0.05% trypsin containing 0.02% ethylenediamine-tetraacetic acid (EDTA) in PBS for 5 min at 37 °C.

### 4.3. CWR-R1ca Cell Transfection

Cells were seeded in 12-well plates until they reached 60–70% confluency; then a small hairpin RNA (shRNA)-plasmid specific to PCSK9 (Santa Cruz Biotechnology, Dallas, TX, USA) was used for cell transfection. Briefly, first, we prepared the transfection master mixture by adding 0.5 µg lentivirus vector via mixing Lipofectamine 2000 transfection reagent (Carlsbad, CA, USA) with 100 μL Opti- MEM reduced serum media from Gibco (Gaithersburg, MD, USA) for 20 min at room temperature. While we were waiting, media was aspirated, and cells were washed with PBS. Then, 100 μL of Opti-MEM media with or without lipofectamine/plasmid mixture were added to each well and incubated for 4–6 h. Later, the media was aspirated and replaced with a complete serum medium for 2 days. Transfection efficiency was examined by Western blot analysis.

### 4.4. Luciferase-Labeled Cells Lentivirus Transduction

Cells were seeded in 12-well plates. When cells reached 60% confluency, lentiviral particles carrying luciferase from Kerafast (Boston, MA, USA) were transduced into the cells. Briefly, first, we prepared a lentivirus vector with Opti-MEM reduced serum media (1.5 μL/100 μL) and mixed it very gently on ice. Later, media was aspirated, and cells were washed with PBS. Then, 100 μL of Opti-MEM media with or without viral particles were added to each well and incubated for 4–6 h. Later, the media was aspirated and replaced with complete serum media for 2 days. Next, 15 μg/mL of puromycin (Santa Cruz Biotechnology, Dallas, TX, USA) was used for the selection and maintenance of cells expressing the luciferase. Media containing puromycin were replaced every other day. Cellular luciferase activity was measured by adding 25 μL of XenoLight D-luciferin K^+^ salt bioluminescent substrate, PerkinElmer, at a dose of 150 mg/kg in PBS into each well and incubated for 5 min at room temperature [41]. Cells were then imaged using a bioluminescence imaging system (PerkinElmer’s IVIS imaging platform) [41,42].

### 4.5. Cell Proliferation Assay

Cells were seeded in 96-well plates at a density of 5 × 10^3^ cells/well (6 replicate/group) in 10% FBS RPMI-1640 media and left to attach overnight. Next, cells were treated with different PS concentrations (reconstituted in 25 mM DMSO) or vehicle control (VC) for 72 h. At the end of the treatment duration, the viable cell number was quantified using a 3-(4,5-dimethylthiazolyl-2)-2,5-diphenyltetrazolium bromide (MTT, VWR International, Suwanee, GA, USA) assay as described earlier [20,43]. A long-term viability assay was conducted as cells seeded with a few numbers of cells at a rate of 1 × 10^3^ cells/well and treated with different PS concentrations for 10 days [40].

### 4.6. Cell Migration Wound Healing Scratch Assay

Cells were seeded in sterile flat-bottom 24-well plates (3 replicates/group) and allowed to form a sub-confluent cell monolayer per well overnight. Wounds were then scratched in each cell monolayer using a 200 µL-sterile pipette tip. Media was removed, and cells were washed twice with PBS to remove floating cells. CWR-R1ca cells were incubated with culture media containing different doses of PS or VC in 10% serum-containing media for 24 h or until the wound was closed in VC wells. Media was removed, and cells were washed with pre-cooled PBS, fixed with methanol previously cooled to −20 °C, stained with Giemsa for 5 min, and rinsed 5 times with 2 mL of tap water. Imaging capture and distant migration were calculated as previously reported [20,42,43].

### 4.7. Colony Formation Assay

Cells were seeded in a 6-well plate at a density of 1 × 10^3^ cells per well and cultured overnight. Different PS concentrations were added to 400 µL media. Fresh cell culture medium was added to the wells every 3 days. The plate was incubated for 12 days. Later, media was removed, and cells were washed with pre-cooled PBS, fixed with methanol previously cooled to −20 °C, stained with Giemsa for 5 min, and rinsed 5 times with 2 mL of tap water. The images of well plates were taken using a digital camera, and the colonies were counted using ImageJ software [59].

### 4.8. Western Blot Analysis

Cells were homogenized and lysed in radioimmunoprecipitation assay (RIPA) buffer (Qiagen Sciences Inc., Valencia, CA, USA) and cleared by centrifugation. Animal tumor tissues were homogenized with a 1× protease inhibitor and cleared by centrifugation. Samples were resolved on 8% sodium dodecyl sulfate-polyacrylamide gel electrophoresis (SDS-PAGE) gels and transferred to polyvinylidene difluoride membranes, and Western blotting analysis was performed as previously described [20,36,42,43]. Actin presented in the images represents the loading protein control for one or more markers from the same cell lysates. Chemiluminescence was used to detect the positive bands on the membrane. [20,36,42,43].

### 4.9. Tissue Microarray Immunohistochemistry and Histochemical Score

A paraffin-embedded tissue microarray (TMA) slide comprised of 64 human cases with 192 tissue cores comprising 58 PC tissue specimens, and 6 normal and hyperplastic prostate tissues were purchased from US Biomax with available clinical information (US Biomax, Inc., Derwood, MD). Immunohistochemical (IHC) analysis was performed according to our prior study [60]. Briefly, 3 PC tissue slides (2 slides used as positive controls and the third one as a negative control) and TMA tissue slides were de-waxed in two series of xylene and rehydrated in a descending series of ethanol. The slides were then heated in ethylenediaminetetraacetic acid (EDTA) buffer (pH 8.0) for 25 min. The slides kept at room temperature to cool down. Then, the tissue sections were incubated in a 3% H_2_O_2_ solution to block the endogenous peroxidases. The tissue slides were washed and incubated with rabbit anti-PCSK9 polyclonal antibody (Proteintech, Rosemont, IL) overnight at 4 °C. The developed antigen-antibody complex was identified using VECTASTAIN Elite ABC HRP Kit (Vector Laboratories, Burlingame, CA, USA). The tissue slides were then counterstained with hematoxylin and mounted with a mounting medium. The developed signals were acquired by a Nikon Eclipse 80i light microscope (Nikon Ins., Melville, NY, USA). The immunohistochemical score (IHS) was calculated as we previously described [61]. Briefly, the intensity of the developed signal was scored from zero to 3, and the proportion of distribution was scored from 1 to 5 with a maximum total score of 8.

### 4.10. In Vivo Studies

#### 4.10.1. Animals

Mice were acclimated to pathogen-free conditions in the animal housing facility with free access to drinking water and pelleted rodent chow, namely Teklad S-2335 Mouse Breeder Sterilizable Diet (total fat 11.4%, crude proteins 17.2%, and carbohydrates 45.2%) or Teklad LM-485 (total fat 5.8%, crude proteins 19.1%, and carbohydrates 44.3%, Envigo, Indianapolis, IN, USA). Animals were housed in group cages, with 5 animals per experimental group.

#### 4.10.2. Diet Impact on CWR-R1ca-Luc Cells Tumor Progression and Metastasis

Eight male athymic nude mice (Foxn^1nu^/Foxn^1+^, 5–6 weeks old) (Envigo: Indianapolis, IN, USA) were divided into 2 groups (*n* = 4 each). Group 1 was fed a high-fat diet (HFD) (Teklad S-2335, 11% fat content) or a regular chow diet (Teklad LM-485, 5% fat content). One week later, all mice were subcutaneously (sc) xenografted with 2 × 10^6^ CWR-R1ca-Luciferase transfected (CWR-R1ca-Luc) cells at the suprascapular region. Once tumors started to be palpable, live animal bioluminescence was measured by imaging 2% isoflurane-anesthetized mice using an IVIS Lumina Series III (PerkinElmer) imaging system after intraperitoneal (ip) injection with D-luciferin (XenoLight D-luciferin K^+^ salt bioluminescent Substrate, PerkinElmer) at a dose of 150 mg/kg per animal in sterile PBS [33,34]. The photons emitted from luciferase-expressing cells within the animal body and transmitted through the tissue quantified using the Living Image software program (PerkinElmer). Images representing light intensity (blue, least intense, and red, most intense) were generated and quantified as photons/second [41,42,43]. Live animals were imaged and bioluminescence was recorded once a week to monitor the tumor progression. The animals’ health status was monitored routinely for weight loss or any signs of altered behavioral or motor ability while in their cages. Tumor volume was monitored every three days with a digital caliber (VWR, Radnor, PA, USA). The experiment terminated after 8 weeks of primary tumor surgical excision. Isoflurane anesthetized mice were then sacrificed by cervical dislocation according to the approved IACUC protocol. Prostate tumors and body organs (brain, heart, lung, liver, kidney and spleen) of all animals were collected, weighted, imaged, and bioluminescence imaged to monitor distant organ metastasis. Blood was collected for quantification analysis. All data are presented as the mean ± SEM. Statistical differences were evaluated by Student’s t-test analysis with two groups, and the criterion for statistical significance was *p* < 0.05.

#### 4.10.3. Recurrence Study

Ten male mice fed an HFD were xenografted with 2 × 10^6^ CWR-R1ca-Luc cells at the suprascapular region. Mice were monitored and bioluminescence imaged for tumor progression. Once the tumor reached 100 mm^3^, mice were randomized to two groups, *n* = 5 each: (i) VC and (ii) PS-treated groups. PS treatment was administered orally using a flexible plastic VWR gavage tube (2 mm diameter gavage tube with a stainless-steel bite protector, 18-gauge, 3.81 cm long) at 10 mg/kg dissolved in 0.2% DMSO in PBS. The VC group received equivalent volumes of 0.2% DMSO/PBS. Treatments continued daily until the experiment ended. One week later, mice tumors were surgically excised, at which point the average tumor volume then was ~200 mm^3^. Mice were imaged immediately after surgery to confirm excision operation efficiency by assuring complete removal of the bioluminescent tumor mass. The experiment continued for additional eight weeks after resection surgery, with once-a-week live animal bioluminescence imaging to monitor the progression of tumor locoregional and distant recurrences. Mice were sacrificed at the study end; tumors were harvested, and organs were weighed and imaged to semi-quantify distant recurred tumor bioluminescence in each organ.

### 4.11. Serum Cholesterol Levels

Mice serum samples were prepared from fresh blood samples collected at the study’s end. Samples were analyzed for serum cholesterol, following the manufacturer’s protocol using the Cell Biolabs colorimetric assay kit (Cell Biolabs Inc, San Diego, CA, USA) [20,36].

### 4.12. Serum LDL-C Levels

Mice serum LDL-C levels were measured using a Crystal Chem mouse LDL-C assay kit (Cat. No. 79980) according to the manufacturer’s protocol. Briefly, 3 µL of serum samples were added to 225 µL of PVS/PEGME enzyme solution and incubated at 37 °C for 5 min. Then, 75 μL of the de-complexing agent was added and re-intubated for another 5 min at 37 °C. Later, optical density (OD) was measured at 600 nm wavelength.

### 4.13. Serum PCSK9 Levels

Mice serum PCSK9 levels were measured using a Rockland ELISA kit (Rockland Immunochemicals, Inc. Limerick, PA, USA, Cat. No. KOA0660) according to the manufacturer’s protocol.

### 4.14. Serum Prostate-Specific Antigen (PSA) Level

Mice serum PSA levels were measured using an enzyme-linked immunosorbent assay (ELISA) kit (MYBIOSOURCE, San Diego, CA, USA, Cat. No. MBS034265) according to the manufacturer’s protocol. Briefly, a 50 µL sample was added to each well, followed by 100 µL HRP-conjugate reagent, and incubated for 1 h at 37 °C. Then, 50 µL of each chromogen solution A and B were added to each well and incubated for 15 min. Later, the reaction was stopped by adding 50 µL of stop solution. OD was read at λ450 nm using an ELISA plate reader (BioTek, Winooski, VT, USA).

### 4.15. Statistical Analysis

Statistical analysis was performed using the one-way ANOVA followed by Dunnett’s test and Student’s *t*-test (when comparing just two experimental groups). The number of experiments given in each figure in captions and all data presented in this study are expressed as mean ±SEM. All statistical tests were performed using the GraphPad Prism Version 9.4.1 software (GraphPad Software Inc., La Jolla, CA, USA), and a probability value of <0.05 was considered significant (*, *p* < 0.05; **, *p* < 0.01; ***, *p* ≤ 0.001; and ****, *p* < 0.0001). GraphPad Prism was used to compare between two experimental groups, and unpaired Student’s *t*-tests were applied for TMA data.

### 4.16. Ethical Considerations

All animal experiments were preapproved by the University of Louisiana at Monroe Institutional Animal Care and Use Committee (IACUC) and conducted in strict accordance with good animal practice as defined by the NIH guidelines (Protocol #21MAY-KES-01).

## 5. Conclusions

The current study assures the relevance of PCSK9 and HFD for exaggerated PC pathogenesis. PCSK9 was identified as a critical effector for mCRPC motility. Current therapies have limited cancer recurrence preventive activity and are unlikely to eradicate residual quiescent tumor cells. PS showed selective cytostatic effects on tumor cells without direct cytotoxicity, minimizing any potential for off-target side effects. PS significantly reduced PCSK9 expression level in vitro and in collected type II ADI CRPC tumors, suggesting possible dual extracellular and intracellular pathway of action, unlike the current humanized FDA-approved mAbs, which only act extracellularly since their large molecular size precludes tumor cells entry. PS is privileged over current mAbs because it is orally active, dually suppresses the PCSK9 expression and PPIs, is cost-effective through a sustained fermentation supply source, has readily scalable preparation methods, and possesses a drug-like unique optimal molecular size and rare pharmacophoric features. The study validated the marine natural product PS as a novel small molecule lead for the control of mCRPC recurrence through targeting PCSK9 expression and binding with LDLR.

## Data Availability

No publicly archived datasets were analyzed or generated during the study.

## Supplementary Materials

The following supporting information can be downloaded at: https://www.mdpi.com/article/10.3390/md21040215/s1, Table S1: Clinical characteristics of prostate cancer tissue cores.

## References

[1] Karantanos T., Corn P.G., Thompson T.C. (2013). Prostate cancer progression after androgen deprivation therapy: Mechanisms of castrate resistance and novel therapeutic approaches. Oncogene.

[2] Labrecque M.P., Coleman I.M., Brown L.G., True L.D., Kollath L., Lakely B., Nguyen H.M., Yang Y.C., da Costa R.M.G., Kaipainen A. (2019). Molecular profiling stratifies diverse phenotypes of treatment-refractory metastatic castration-resistant prostate cancer. J. Clin. Investig..

[3] Bluemn E.G., Coleman I.M., Lucas J.M., Coleman R.T., Hernandez-Lopez S., Tharakan R., Bianchi-Frias D., Dumpit R.F., Kaipainen A., Corella A.N. (2017). Androgen receptor pathway-independent prostate cancer is sustained through FGF signaling. Cancer Cell.

[4] Ma C., Yoshioka M., Boivin A., Gan L., Takase Y., Labrie F., St-Amand J. (2009). Atlas of dihydrotestosterone actions on the transcriptome of prostate in vivo. Prostate.

[5] Montgomery R.B., Mostaghel E.A., Vessella R., Hess D.L., Kalhorn T.F., Higano C.S., True L.D., Nelson P.S. (2008). Maintenance of intratumoral androgens in metastatic prostate cancer: A mechanism for castration-resistant tumor growth. Cancer Res..

[6] Locke J.A., Guns E.S., Lubik A.A., Adomat H.H., Hendy S.C., Wood C.A., Ettinger S.L., Gleave M.E., Nelson C.C. (2008). Androgen levels increase by intratumoral de novo steroidogenesis during progression of castration-resistant prostate cancer. Cancer Res..

[7] Mostaghel E.A., Nelson P.S. (2008). Intracrine androgen metabolism in prostate cancer progression: Mechanisms of castration resistance and therapeutic implications. Best Practice Res. Clin. Endocr. Metab..

[8] Bryce A., Ryan C.J. (2011). Development and clinical utility of abiraterone acetate as an androgen synthesis inhibitor. Clin. Pharmacol. Ther..

[9] Cheng H.H., Gulati R., Azad A., Nadal R., Twardowski P., Vaishampayan U.N., Agarwal N., Heath E.I., Pal S.K., Rehman H.T. (2015). Activity of enzalutamide in men with metastatic castration-resistant prostate cancer is affected by prior treatment with abiraterone and/or docetaxel. Prost. Can. Prost. Dis..

[10] Guerrero J., Alfaro I.E., Gómez F., Protter A.A., Bernales S. (2013). Enzalutamide, an androgen receptor signaling inhibitor, induces tumor regression in a mouse model of castration-resistant prostate cancer. Prostate.

[11] Mostaghel E.A., Marck B.T., Plymate S.R., Vessella R.L., Balk S., Matsumoto A.M. (2011). Nelson PS, Montgomery RB. Resistance to CYP17A1 inhibition with abiraterone in castration-resistant prostate cancer: Induction of steroidogenesis and androgen receptor splice variants. Clin. Cancer Res..

[12] Siegel R.L., Miller K.D., Wagle N.S., Jemal A. (2023). Cancer statistics, 2023. CA Cancer J. Clin..

[13] Ikonen E. (2008). Cellular cholesterol trafficking and compartmentalization. Nat. Rev. Mol. Cell Biol..

[14] Simons K. (2000). How cells handle cholesterol. Science.

[15] Di Vizio D., Solomon K.R., Freeman M.R. (2008). Cholesterol and cholesterol-rich membranes in prostate cancer: An update. Tumori.

[16] Freeman M.R., Solomon K.R. (2003). Cholesterol and prostate cancer. J. Cell. Biochem..

[17] Vance J.E., Vance D. (1996). Biochemistry of Lipids, Lipoproteins and Membranes.

[18] Brown M.S., Goldstein J.L. (1986). Receptor-mediated pathway for cholesterol homeostasis. Science.

[19] Seidah N.G., Prat A. (2012). The biology and therapeutic targeting of the proprotein convertases. Nat. Rev. Drug Discov..

[20] Abdelwahed K.S., Siddique A.B., Mohyeldin M.M., Qusa M.H., Goda A.A., Singh S.S., Ayoub N.M., King J.A., Jois S.D., El Sayed K.A. (2020). Pseurotin A as a novel suppressor of hormone dependent breast cancer progression and recurrence by inhibiting PCSK9 secretion and interaction with LDL receptor. Pharmacol. Res..

[21] Sun X., Essalmani R., Day R., Khatib A.M., Seidah N.G., Prat A. (2012). Proprotein convertase subtilisin/kexin type 9 deficiency reduces melanoma metastasis in liver. Neoplasia.

[22] Bonaventura A., Grossi F., Montecucco F. (2020). PCSK9 is a promising prognostic marker in patients with advanced NSCLC. Cancer Immunol. Immunother..

[23] Liu X., Bao X., Hu M., Chang H., Jiao M., Cheng J., Xie L., Huang Q., Li F., Li C.Y. (2020). Inhibition of PCSK9 potentiates immune checkpoint therapy for cancer. Nature.

[24] Xu B., Li S., Fang Y., Zou Y., Song D., Zhang S., Cai Y. (2021). Proprotein convertase subtilisin/kexin type 9 promotes gastric cancer metastasis and suppresses apoptosis by facilitating MAPK signaling pathway through HSP70 up-regulation. Front. Oncol..

[25] Azhar S., Reaven E. (2002). Scavenger receptor class BI and selective cholesteryl ester uptake: Partners in the regulation of steroidogenesis. Mol. Cell. Endocr..

[26] Krycer J.R., Kristiana I., Brown A.J. (2009). Cholesterol homeostasis in two commonly used human prostate cancer cell-lines, LNCaP and PC-3. PLoS ONE.

[27] Leon C.G., Locke J.A., Adomat H.H., Etinger S.L., Twiddy A.L., Neumann R.D., Nelson C.C., Guns E.S., Wasan K.M. (2010). Alterations in cholesterol regulation contribute to the production of intratumoral androgens during progression to castration-resistant prostate cancer in a mouse xenograft model. Prostate.

[28] Sekine Y., Koike H., Nakano T., Nakajima K., Takahashi S., Suzuki K. (2009). Remnant lipoproteins induced proliferation of human prostate cancer cell, PC-3 but not LNCaP, via low density lipoprotein receptor. Cancer Epidemiol..

[29] Wang L., Li S., Luo H., Lu Q., Yu S. (2022). PCSK9 promotes the progression and metastasis of colon cancer cells through regulation of EMT and PI3K/AKT signaling in tumor cells and phenotypic polarization of macrophages. J. Experim. Clin. Cancer Res..

[30] Kaleem S., Qin L., Yi W., Lian X.-Y., Zhang Z. (2020). Bioactive metabolites from the Mariana Trench sediment-derived fungus *Penicillium* sp. SY2107. Mar. Drugs.

[31] Han J., Liu M., Jenkins I.D., Liu X., Zhang L., Quinn R.J., Feng Y. (2020). Genome-inspired chemical exploration of marine fungus *Aspergillus fumigatus* MF071. Mar. Drugs.

[32] Anjum K., Bi H., Chai W., Lian X.Y., Zhang Z. (2018). Antiglioma pseurotin A from marine *Bacillus* sp. FS8D regulating tumour metabolic enzymes. Nat. Prod. Res..

[33] Lee M.S., Wang S.W., Wang G.J., Pang K.L., Lee C.Q., Kuo Y.H., Cha H.J., Lin R.K., Lee T.H. (2016). Angiogenesis inhibitors and anti-Inflammatory agents from *Phoma* sp. NTOU4195. J. Nat. Prod..

[34] Uchoa P.K.S., Pimenta A.T.A., Braz-Filho R., de Oliveira M.C.F., Saraiva N.N., Rodrigues B.S.F., Pfenning L.H., Abreu L.M., Wilke D.V., Florêncio K.G.D. (2017). New cytotoxic furan from the marine sediment-derived fungi *Aspergillus niger*. Nat. Prod. Res..

[35] Copmans D., Rateb M., Tabudravu J.N., Pérez-Bonilla M., Dirkx N., Vallorani R., Diaz C., del Palacio J.P., Smith A.J., Ebel R. (2018). Zebrafish-based discovery of antiseizure compounds from the Red Sea: Pseurotin A2 and azaspirofuran A. ACS Chem. Neurosci..

[36] Abdelwahed K., Siddique A.B., Qusa M., King J.A., Souid S., Abd Elmageed Z., El Sayed K.A. (2021). The PCSK9 axis-targeting pseurotin A as a novel prostate cancer recurrence suppressor lead. ACS Pharmacol. Translat. Sci..

[37] McGehee O.C., Ebrahim H.Y., Rad A.H., Abdelwahed K.S., Mudhish E.A., King J.A., Helal I.E., Meyer S.A., El Sayed K.A. (2023). Towards developing novel prostate cancer recurrence suppressors: Acute toxicity of pseurotin A, an orally active PCSK9 axis-targeting small-molecule in Swiss albino mice. Molecules.

[38] Litvinov I.V., Antony L., Dalrymple S.L., Becker R., Cheng L., Isaacs J.T. (2006). PC3, but not DU145, human prostate cancer cells retain the coregulators required for tumor suppressor ability of androgen receptor. Prostate.

[39] Shourideh M., DePriest A., Mohler J.L., Wilson E.M., Koochekpour S. (2016). Characterization of fibroblast-free CWR-R1ca castration-recurrent prostate cancer cell line. Prostate.

[40] Riss T.L., Moravec R.A. (2004). Use of multiple assay endpoints to investigate the effects of incubation time, dose of toxin, and plating density in cell-based cytotoxicity assays. Assay Drug Dev. Technol..

[41] Clark A.J., Fakurnejad S., Ma Q., Hashizume R. (2016). Bioluminescence imaging of an immunocompetent animal model for glioblastoma. J. Vis. Exp..

[42] Siddique A.B., Kilgore P.C.S.R., Tajmim A., Singh S.S., Meyer S.A., Jois S.D., Cvek U., Trutsch M., El Sayed K.A. (2020). (−)-Oleocanthal as a dual c-MET-COX2 inhibitor for the control of lung cancer. Nutrients.

[43] Siddique A., Ebrahim H.Y., Tajmim A., King J., Abdelwahed K., Abd Elmageed Z.Y., El Sayed K.A. (2022). Oleocanthal attenuates metastatic castration-resistant prostate cancer progression and recurrence by targeting SMYD2. Cancers.

[44] Awasthi S., Gerke T., Park J.Y., Asamoah F.A., Williams V.L., Fink A.K., Balkrishnan R., Lee D.I., Malkowicz S.B., Lal P. (2019). Optimizing time to treatment to achieve durable biochemical disease control after surgery in prostate cancer: A multi-institutional cohort study. Cancer Epidemiol. Biomark. Prev..

[45] Pelton K., Freeman M.R., Solomon K.R. (2012). Cholesterol and prostate cancer. Current Opin. Pharmacol..

[46] Jamnagerwalla J., Howard L.E., Allott E.H., Vidal A.C., Moreira D.M., Castro-Santamaria R., Andriole G.L., Freeman M.R., Freedland S.J. (2018). Serum cholesterol and risk of high-grade prostate cancer: Results from the REDUCE study. Prostate Cancer Prostatic Dis..

[47] Murtola T.J., Kasurinen T.V., Talala K., Taari K., Tammela T.L., Auvinen A. (2019). Serum cholesterol and prostate cancer risk in the Finnish randomized study of screening for prostate cancer. Prostate Cancer Prostatic Dis..

[48] Stopsack K.H., Gerke T.A., Andrén O., Andersson S.O., Giovannucci E.L., Mucci L.A., Rider J.R. (2017). Cholesterol uptake and regulation in high-grade and lethal prostate cancer. Carcinogenesis.

[49] Mohler J.L., Gregory C.W., Ford O.H., Kim D., Weaver C.M., Petrusz P., Wilson E.M., French F.S. (2004). The androgen axis in recurrent prostate cancer. Clin. Cancer Res..

[50] Mostaghel E.A., Solomon K.R., Pelton K., Freeman M.R., Montgomery R.B. (2012). Impact of circulating cholesterol levels on growth and intratumoral androgen concentration of prostate tumors. PLoS ONE.

[51] Narita S., Nara T., Sato H., Koizumi A., Huang M., Inoue H.T. (2019). Research evidence on high-fat diet-induced prostate cancer development and progression. J. Clin. Med..

[52] Paciullo F., Fallarino F., Bianconi V., Mannarino M.R., Sahebkar A., Pirro M. (2017). PCSK9 at the crossroad of cholesterol metabolism and immune function during infections. J. Cell. Physiol..

[53] Maxwell K.N., Fisher E.A., Breslow J.L. (2005). Overexpression of PCSK9 accelerates the degradation of the LDLR in a post-endoplasmic reticulum compartment. Proc. Nat. Acad. Sci. USA.

[54] Pettersen D., Fjellström O. (2018). Small molecule modulators of PCSK9–A literature and patent overview. Bioorg. Med. Chem. Lett..

[55] Fang S., Yarmolinsky J., Gill D., Bull C.J., Perks C.M., Smith G.D., Gaunt T.R., Richardson T.G., The PRACTICAL Consortium (2023). Genetically proxied PCSK9 inhibition provides indication of lower prostate cancer risk: A Mendelian randomization study. PLoS Med..

[56] Athavale D., Chouhan S., Pandey V., Mayengbam S.S., Singh S., Bhat M.K. (2018). Hepatocellular carcinoma-associated hypercholesterolemia: Involvement of proprotein-convertase-subtilisin-kexin type-9 (PCSK9). Cancer Metab..

[57] Obenauf A.C., Massagué J. (2015). Surviving at a distance: Organ-specific metastasis. Trends Cancer.

[58] Nguyen D.X., Bos P.D., Massagué J. (2009). Metastasis: From dissemination to organ-specific colonization. Nat. Rev. Cancer.

[59] https://imagej.net/ij/index.html.

[60] Gaballah M.S.A., Ali H.E.A., Hassan Z.A., Mahgoub S., Ali H.I., Rhim J.S., Zerfaoui M., El Sayed K.A., Stephen D., Sylvester P.W. (2022). Small extracellular vesicle-associated miR-6068 promotes aggressive phenotypes of prostate cancer through miR-6068/HIC2/SIRT1 axis. Am. J. Cancer Res..

[61] Abd Elmageed Z.Y., Moroz K., Srivastav S.K., Fang Z., Crawford B.E., Moparty K., Thomas R., Abdel-Mageed A.B. (2013). High circulating estrogens and selective expression of ER-beta in prostate tumors of Americans: Implications for racial disparity of prostate cancer. Carcinogenesis.

