# Supplementary material for: Pseurotin A Validation as a Metastatic Castration-Resistant Prostate Cancer Recurrence-Suppressing Lead via PCSK9-LDLR Axis Modulation
Source: Mar Drugs. 2023 Mar 28;21(4):215. doi: 10.3390/md21040215 (PMC10144979; doi:10.3390/md21040215)
Supplement: Supplementary file 1 [file marinedrugs-21-00215-s001.zip › marinedrugs-2277965-supplementary.pdf]

marine drugs

Supplementary Table S1: Clinical characteristics of prostate cancer tissue cores.

Variable	Normal	PC
**Total number**	18	174
**Age (Mean ± SD)**	35.3 ± 5.7	68.7 ± 9.1
**Gleason score (n)**		
**GS <7**		55
**GS=7**		47
**GS>7**		52
**Not reported**		20
**T-stage (n)**		
**I**		0
**II**		132
**III**		39
**IV**		3
**Lymph node involvement (n)**		
**Yes**		3
**No**		171
